# Prosthetic Embodiment and Body Image Changes in Patients Undergoing Bionic Reconstruction Following Brachial Plexus Injury

**DOI:** 10.3389/fnbot.2021.645261

**Published:** 2021-04-30

**Authors:** Agnes Sturma, Laura A. Hruby, Anna Boesendorfer, Anna Pittermann, Stefan Salminger, Clemens Gstoettner, Olga Politikou, Ivan Vujaklija, Dario Farina, Oskar C. Aszmann

**Affiliations:** ^1^Clinical Laboratory for Bionic Extremity Reconstruction, Department of Surgery, Medical University of Vienna, Vienna, Austria; ^2^Neurorehabilitation Engineering Group, Department of Bioengineering, Imperial College London, London, United Kingdom; ^3^Department of Orthopedics and Trauma Surgery, Medical University of Vienna, Vienna, Austria; ^4^Department of Clinical Psychology, General Hospital of Vienna, Vienna, Austria; ^5^Division of Plastic and Reconstructive Surgery, Department of Surgery, Medical University of Vienna, Vienna, Austria; ^6^Bionic and Rehabilitation Engineering Research Group, Department of Electrical Engineering and Automation, Aalto University, Espoo, Finland

**Keywords:** brachial plexus injury, bionic reconstruction, human-machine interfaces, upper limb amputation, prosthesis, body image, embodiment/bodily experience

## Abstract

Brachial plexus injuries with multiple-root involvement lead to severe and long-lasting impairments in the functionality and appearance of the affected upper extremity. In cases, where biologic reconstruction of hand and arm function is not possible, bionic reconstruction may be considered as a viable clinical option. Bionic reconstruction, through a careful combination of surgical augmentation, amputation, and prosthetic substitution of the functionless hand, has been shown to achieve substantial improvements in function and quality of life. However, it is known that long-term distortions in the body image are present in patients with severe nerve injury as well as in prosthetic users regardless of the level of function. To date, the body image of patients who voluntarily opted for elective amputation and prosthetic reconstruction has not been investigated. Moreover, the degree of embodiment of the prosthesis in these patients is unknown. We have conducted a longitudinal study evaluating changes of body image using the patient-reported Body Image Questionnaire 20 (BIQ-20) and a structured questionnaire about prosthetic embodiment. Six patients have been included. At follow up 2.5–5 years after intervention, a majority of patients reported better BIQ-20 scores including a less negative body evaluation (5 out of 6 patients) and higher vital body dynamics (4 out of 6 patients). Moreover, patients described a strong to moderate prosthesis embodiment. Interestingly, whether patients reported performing bimanual tasks together with the prosthetic hand or not, did not influence their perception of the prosthesis as a body part. In general, this group of patients undergoing prosthetic substitution after brachial plexus injury shows noticeable inter-individual differences. This indicates that the replacement of human anatomy with technology is not a straight-forward process perceived in the same way by everyone opting for it.

## Introduction

**Figure 1:**
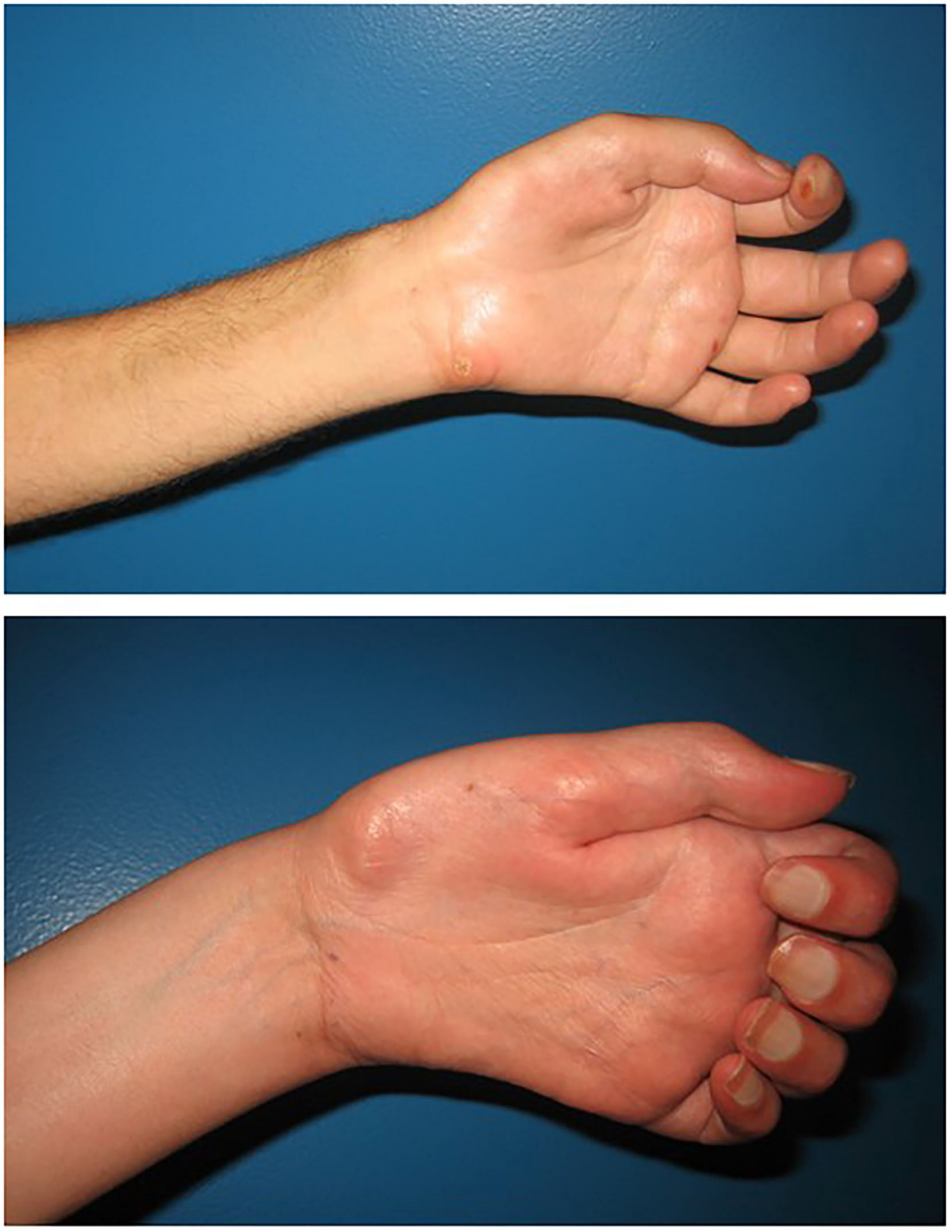
Lateral view of the hands of two different patients after a brachial plexus avulsion, showing different degrees of atrophy and intrinsic stiffness.

Global brachial plexus injuries or lower root avulsions have a devastating impact on upper extremity function and quality of life of affected patients (Carlstedt, 2008; Franzblau and Chung, 2015), who are predominantly young male adults (Seddighi et al., 2016). Due to the loss of neural connectivity of the hand and arm, also referred to as “inner amputation,” both muscle function and sensibility of the affected skin are permanently impaired. In severe cases, this leads to a complete loss of hand function (Franzblau et al., 2015). Traditional surgical procedures, such as nerve grafting, nerve transfers, tendon transfers and arthrodesis, may fail to restore full function, sensation, and comfort with appearance of the affected extremity (Terzis and Papakonstantinou, 2000). Aside from functional impairments and pain caused by the injury, negative psychological consequences of brachial plexus lesions and upper extremity nerve damage have been widely reported (Franzblau et al., 2014; Franzblau and Chung, 2015; Miller et al., 2016). Post-traumatic stress disorder, depression and reduced social participation commonly occur (Bailey et al., 2009; Franzblau et al., 2014). While the reasons for these consequences are several, the changed visual appearance of the upper extremity seems to be a main factor eventually leading to reduced participation in and avoidance of social gatherings. Indeed, recent studies suggest that up to two third of all brachial plexus injury patients do not accept the appearance of their often motionless hand and arm, which can eventually become stiff, cold, and atrophic as shown in Figure 1 (Carlstedt, 2008; Franzblau et al., 2014). This aesthetic dissatisfaction and the distorted body image affect social life, resulting in the reduced willingness to participate in social activities, especially if these include meeting new people (Mancuso et al., 2015; Verma et al., 2019). In the authors' experience the situation over time tends to gradually worsen, often leaving patients and their immediate social environment frustrated. For selected patients where the aesthetics of the functionless hand is a main concern, radiocarpal and finger joint arthrodeses are an option for improving function, appearance, and quality of life (Giuffre et al., 2012).

In cases of multiple root injury where above mentioned biological treatment options fail to provide sufficient improvement or are not feasible, the recently introduced concept of bionic reconstruction has expanded the treatment possibilities (Aszmann et al., 2015; Maldonado et al., 2016). In this procedure, the functionless hand is amputated and substituted with a myoelectric prosthesis. A psychological and functional assessment before amputation ensure that patients understand the consequences of the procedure, have the cognitive and emotional prerequisites for decision making, and only receive the intervention if a good prosthetic outcome can be expected (Hruby et al., 2018; Sturma et al., 2018). Prior to amputation, surgical augmentation of the residual limb may be necessary, in order to improve the position of the arm and provide sufficient EMG signals for myoelectric control (Aszmann et al., 2015). While improvements in patients' hand function, quality of life, and perceived disability have been observed (Aszmann et al., 2015; Hruby et al., 2017, 2019a), the long-term impact on body image is still unknown. Furthermore, outcomes in terms of prosthetic embodiment in this unique patient group have not been investigated. These insights into patients' perceptions treated with the novel approach of bionic reconstruction are particularly interesting, given that they have voluntarily opted for an amputation and a prosthetic fitting, which is not the case for the majority of prosthetic users. Understanding the impact of bionic reconstruction on body image perception will thereby offer valuable insights for all fields in medicine where human body function is replaced by technological means.

Therefore, the aim of this study was to investigate the body image of patients after a severe multilevel brachial plexus injury, and to report long-term outcomes after prosthetic fitting, with particular focus on the embodiment of the fitted device.

## Materials and Methods

### Participants

For the purpose of this study, six patients who underwent bionic reconstruction were recruited between the years 2015 to 2018. General inclusion criteria to undergo bionic reconstruction can be found elsewhere (Hruby et al., 2018). Exclusion criteria included injuries or co-morbidities of the central nervous system (CNS), untreated psychological disorders, and patients who had obtained useful hand function after biological reconstruction or who had regained any useful sensory function of the hand. Patient characteristics can be found in Table 1. All patients involved in this study suffered a traumatic multi-level brachial plexus injury, meeting both of the following conditions: (1) damage to upper and lower brachial plexus roots with clinically evident impairment of shoulder, elbow and hand function as well as (2) avulsion of multiple roots confirmed via imaging and/or surgical exploration. The amputation level was determined by the presence of EMG signals in the forearm and sufficient elbow function. In patients where no elbow flexion against resistance could be achieved by surgical means or training, and where no EMG signals could be generated in the forearm, an amputation level above the elbow was chosen to allow prosthetic function (Hruby et al., 2019a).

**Table 1:** Characteristics of included patients.

**Patient ID**	**Type of brachial plexus injury**	**Gender**	**Time between injury and intervention (years)**	**Age group at intervention**	**Time between intervention and long-term follow-up (years)**	**Level of amputation**
P1	Postganglionic injury of C5-6, avulsion of C7, C8-T1 unclear without any clinical function	Male	7	15–24	2.5	Transradial
P2	Avulsion of C5-T1	Male	8.5	55–64	3.5	Transhumeral
P3	Postganglionic injury of upper roots, avulsion of C8-T1	Male	9	35–44	4	Transradial
P4	Postganglionic injury of upper roots, avulsion of roots C8-T1	Male	14	45–54	4.5	Transradial
P5	Postganglionic injury of C5, avulsion of C6-T1	Male	5	55–64	5	Transradial
P6	Postganglionic injury of C5 without any clinical function, avulsion of C6-T1	Male	21.5	35–44	5	Glenohumeral

*All patients were male and had a brachial plexus injury with multiple root involvement. The presence of elbow flexion against resistance and EMG signals on the forearm was a requirement for an amputation below the elbow. The level of the above-elbow amputations was decided based on the patient's preferences and physical findings such as shoulder stability*.

During the mandatory pre-surgical assessment, all patients mentioned limited hand function as well as aesthetic dissatisfaction as current problems they wished to ease with bionic reconstruction, with function being the dominant motivator. Four of them (P2, P3, P4, P6) also named shoulder pain and/or deafferentation pain as a factor currently limiting their quality of life. Three of the patients (P1, P4, P5) described their lame limb as “hindering” in daily life, and P1 and P5 explicitly expressed that they would even consider an amputation without prosthetic replacement. Understanding the limitations of myoelectric prostheses (such as the lack of sensory feedback, no use in wet surroundings, and function not comparable with a heathy human hand) was a requirement for elective amputation. While P2–5 only expected a moderate functional gain from the prosthesis, P1 expected a clear improvement and P6 originally had some unrealistic expectations that were lowered in discussions with the medical team.

This study was approved by the ethics committee of the Medical University of Vienna, Austria and was carried out in accordance with the standards set by the Declaration of Helsinki (World Medical Association, 2013). All patients provided written informed consent to participate in this study.

### Study Design and Procedure

**Figure 2:**
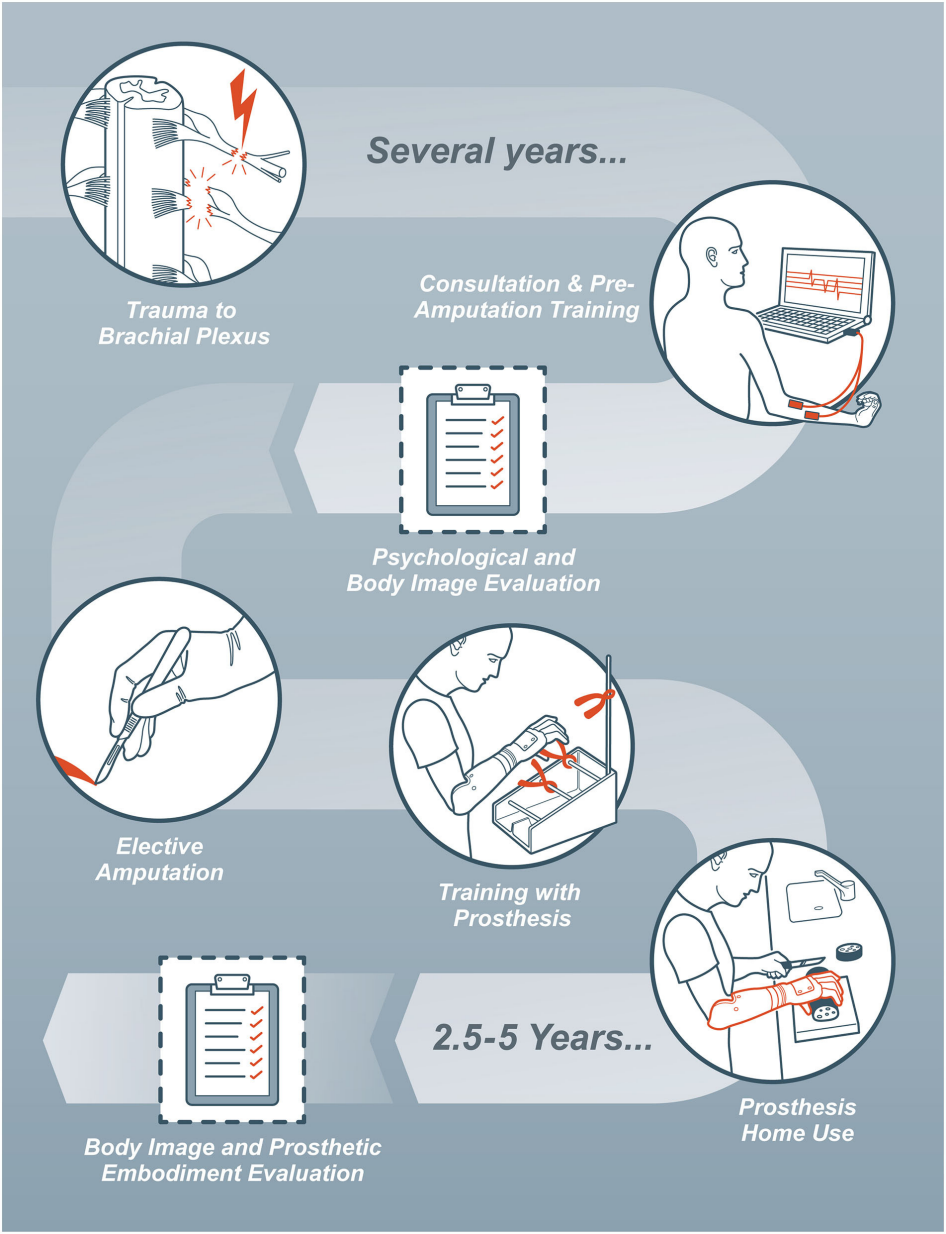
Flowchart of the medical treatment process and assessments performed within this study.

All included patients underwent bionic hand reconstruction according to the latest best practices (Hruby et al., 2017; Sturma et al., 2018). For each of the study participants, previous attempts to restore biological hand function had failed. These patients approached our team with the wish to have their hand replaced with a prosthetic device, or were referred by their physicians. After the first clinical assessment, an experienced therapist (AS) aimed at identifying two independent surface electromyographic (sEMG) signals on the functionless arm. These were meant to provide control inputs for the myoelectric prosthesis following the potential amputation. In patients where no sEMG signals could be detected, free muscle and nerve transfers were considered in order to create an additional neural interface for prosthetic control (Aszmann et al., 2015). Upon appropriate identification of signals, their selective control and stable presentation was trained using biofeedback techniques (Hruby et al., 2019b). If there was an unstable shoulder or weak elbow function in patients with EMG signals on the forearm, this was trained as well. Also, grasp function was trained with a prosthetic device mounted on a table and on the functionless arm. In addition to training, this allowed patients to experience realistic prosthetic function before committing to the amputation procedure. The final decision to undergo elective amputation was made after a psychological assessment conducted by an experienced psychologist (AP) (Hruby et al., 2018). Patients who were deemed suitable and agreed to participate in the study, were asked to fill out the Body Image Questionnaire (BIQ-20), as well as a questionnaire regarding their disability in daily live (Disabilities of Arm Shoulder and Hand, DASH). Post-operatively patients were fitted with a standard myoelectric prosthesis within the first 3 months, and received further prosthetic training. 2.5–5 years after the intervention, patients were asked to repeat the BIQ-20 and the DASH, as well as to answer selected questions described below regarding the embodiment of their prosthetic limb. The study procedure is outlined in Figure 2.

### Assessment Instruments

The participants' body image was evaluated using the Body Image Questionnaire 20 (BIQ-20; originally published in German as “Fragebogen zum Körperbild - FBK-20”) (Clement and Löwe, 1996). The BIQ-20 is a validated 20-item questionnaire designed to evaluate body awareness and possible body image disorders. It consists of two independent scales, negative body evaluation and vital body dynamics. The first includes possible negative associations one might have with their physical appearance and associated well-being (e.g., “There is something wrong with my looks/appearance”). The scale for vital body dynamics summarizes how physically strong and healthy individuals describe themselves (e.g., “I am physically capable of doing many things”) (Löwe and Clement, 1996). An improvement in the body image is thereby seen with a higher score in the vital body dynamics scale and a lower score in the negative body evaluation scale.

Furthermore, at follow-up, participants were asked to report how often they had been wearing their prosthesis within the week prior to the assessment. Moreover, they were presented with six statements related to the embodiment of their prosthesis and were asked to indicate to which extent they can relate to them:

1. “I had the feeling that the prosthesis was part of my body.”
2. “I felt the prosthesis only as a tool, and not as a part of my body.”
3. “I did bimanual tasks with my intact arm/hand together with my prosthesis.”
4. “I felt that I had full control over the prosthesis.”
5. “I liked wearing the prosthesis.”
6. “I felt that my prosthesis looked like a real part of the body.”

For all questions, participants were asked to rate their level of agreement on a Likert-scale from 0 (never) to 10 (always).

In order to understand how participants perceived functional changes in daily life, the patient-reported Disabilities of Arm Shoulder and Hand (DASH) questionnaire was used before and after elective amputation as a secondary outcome (Gummesson and Ekdahl, 2003). Based on the answers to 30 questions, a score from 0 (no functional impairment) to 100 was obtained, with a minimal clinically important difference suggested at 10.83 points (Franchignoni et al., 2014). As patients rate the difficulties they have in daily life - independent from the hand they use for completing tasks - the DASH needs to be seen as a widely-used general assessment instrument for upper limb function rather than an instrument to purely measure prosthetic function.

### Statistical Analysis

For all outcomes, explorative statistics were considered. Since the BIQ-20 delivers data on an ordinal scale, a Wilcoxon test with a significance level of *p* < 0.05 was used to assess differences between baseline and follow-up. Statistical analysis was performed using SPSS 26 (IBM, Armonk, NY, United States).

## Results

### Body Image Questionnaire

The BIQ-20 scores for negative body evaluation and vital body dynamics pre- and post-bionic reconstruction are displayed in Table 2. The single item answers for every participant can be found in the Supplementary Material. As both parameters change with age, Table 2 also presents the age-matched mean values for both scores as a reference. The values originate from a survey of a representative German sample population (*n* = 2,473) as described by Albani et al. (2006). The median value for negative body evaluation improved significantly (*p* = 0.046) from 19 (IQR 16.75–25) at baseline to 14.5 (IQR 12.25–17.5) at follow up with bionic reconstruction. No significant changes were found in vital body dynamics (*p* = 0.916), with a median value of 34 (IQR 27.75–36.5) before amputation and 28.5 (IQR 28–37.25) after prosthetic fitting.

**Table 2:** DASH outcomes and BIQ-20 scores for negative body evaluation and vital body dynamics before and after elective amputation.

**Patient ID**	**P 1**	**P 2**	**P 3**	**P 4**	**P 5**	**P 6**
DASH score before the intervention	8.3	60	63.3	40.8	47.5	49.2
DASH score after the intervention	5	33.3	30.8	43.3	44.2	30
Negative body evaluation before the intervention	19	19	27*	42*	16	15
Negative body evaluation after the intervention	10	16	13	32*	18	12
Age-matched (Albani et al., 2006) mean value (SD) for negative body evaluation	18.3 (7.1)	19.1 (6.4)	19.0 (7.1)	19.0 (6.6)	19.1 (6.4)	19.0 (7.1)/ 19.0 (6.6)^ 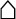 ^
Vital body dynamics before the intervention	37	24*	35	33	26	43
Vital body dynamics after the intervention	40	28	27*	28	29	46
Age-matched (Albani et al., 2006) mean value (SD) for vital body dynamics	39.4 (6.7)	31.8 (7.3)	36.6 (6.8)	34.8 (7.0)	31.8 (7.3)	36.6 (6.8)/34.8 (7.0)^ 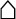 ^

*Please consider that a lower score in the DASH questionnaire and BIQ-20 negative body evaluation is considered a better outcome, while the same is true for a higher value in the vital body dynamics score. An Asterik (*) indicates that the score is worse than the mean ± standard deviation (SD) of the age-matched norm. A blue background with white text in the scores after prosthetic fitting refers to an improvement compared to the assessment before amputation, while an orange background describes a higher value for negative body evaluation, or a lower value for vital body dynamics. ^
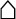
^P6 changed age group between baseline and follow up, with reference values for baseline and follow-up being reported*.

### Prosthetic Embodiment and Prosthesis Wearing Time

When asked about their prosthesis wearing time, three patients (P4, P5, and P6) reported to wear their prosthesis almost daily. One patient wore it every 2nd day (P3), one patient less than twice a week (P2), and one patient (P1) stated that he had not been wearing it within the last week. This patient clarified that he found it easier at work and home to use only his able hand in combination with the residual limb. Still, he enjoys wearing the prosthesis for social events.

**Figure 3:**
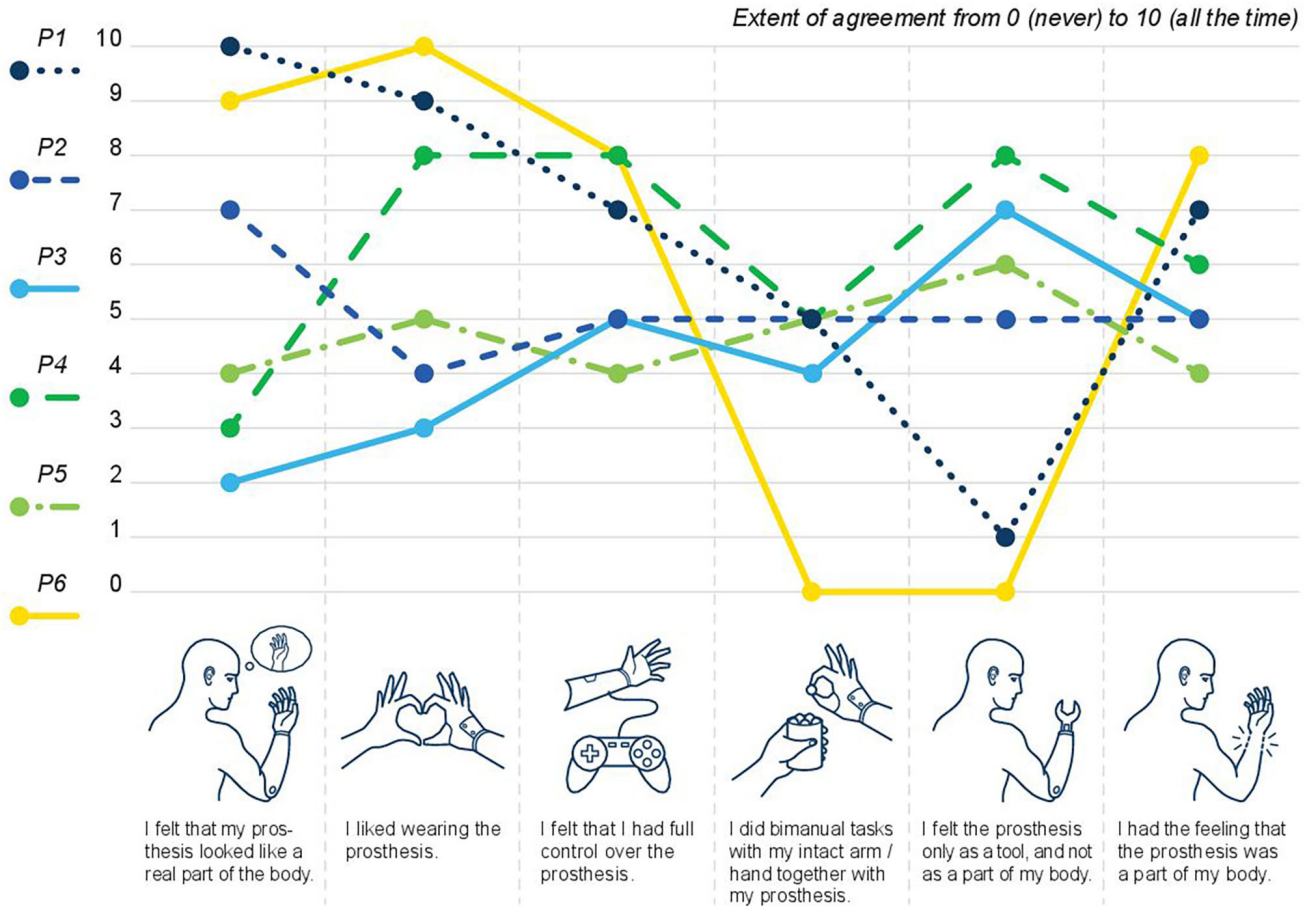
Prosthetic embodiment after bionic reconstruction: the level of agreement from 0 (no agreement/never) to 10 (full agreement/always) is displayed per question and study participant.

The individual ratings of the patients regarding their prosthetic embodiment are displayed in Figure 3, and further summarized in the Supplementary Material. All patients partially or mostly agreed with the statement “I had the feeling that the prosthesis was part of my body” (IQR 5–6.75). Similar results were found for the statement “I felt I had full control over the prosthesis” (IQR 5–7.75). Big inter-individual differences were reported for the statements “I felt that the prosthesis looked like a part of the body” (IQR 3.25–8.5), “I liked wearing the prosthesis” (IQR 4.25–8.75), and “I felt the prosthesis only as a tool and not as a part of my body” (IQR 2–6.75). When asked whether they performed bimanual tasks with their able hand/arm together with their prosthesis, four of six patients rated this with “5,” while the other two had lower ratings of 4 and 0 (IQR 4.25–5).

### Disabilities of Arm Shoulder and Hand

DASH scores of all patients are reported in Table 2. The perceived hand and arm function improved in 5 of 6 participants, with a mean score of 44.9 ± 18.0 at baseline to 31.1 ± 13.0 at follow-up. This represents a clinically important difference (Franchignoni et al., 2014) overall, and a clinically important difference in 3 of 6 patients. The single item ratings for all patients can be found in the Supplementary Material.

## Discussion

Bionic reconstruction is a new technique to restore lost hand function in individuals with severe brachial plexus injuries. The procedure is only considered in patients, where no further improvement of upper limb function can be expected by conventional surgical means or rehabilitative measures (Aszmann et al., 2015). Time between injury and baseline assessment/amputation therefore ranged from 5 to 21.5 years. Thus, the baseline situation evaluated restrictions in body image and functional impairments in patients who have lived with a functionless upper extremity for several years or even decades. This leads to the assumption that they had sufficient time to adapt to the situation, resulting in a relatively stable baseline, which was not expected to change without further interventions. Similarly, at 2.5–5 year follow-up after bionic limb replacement we expected to identify long-lasting effects of bionic reconstruction on patients' body image, not influenced anymore by the initial excitement for the new prosthetic device.

While the evaluation of (prosthetic) hand function in these patients as reported elsewhere (Aszmann et al., 2015; Hruby et al., 2017, 2019a,b) was not an aim of this study, the use of the DASH questionnaire allowed interpretation of body image changes related to hand function in daily life. Hereby, an overall improvement in perceived disability was observed, with five out of six participants reporting better DASH outcomes. Three of them had a clinically meaningful improvement, while no clinically relevant deterioration was reported. This led to the conclusion that perceived overall upper limb function improved with the prosthetic fitting or remained similar. Interestingly, the patient who showed a slightly worse DASH score at follow-up (mainly due to feeling slightly more disabled in some ADLs), also had a decline in *vital body dynamics* in the BIQ-20. In general, when comparing the single-item differences in the DASH score before and after amputation, an overall improvement in performing ADLs (as assessed in questions 1–21), with some inter-individual differences can be observed. DASH results for pain, stiffness and weakness in the remaining arm appear less uniform, with pain during activities even tending to increase after bionic reconstruction. This could be explained by remaining problems related to shoulder instability causing increased pain with the additional weight of the prosthesis and prolonged use of the arm.

In this regard, it is important to note that our study population still had mostly unchanged impairments regarding elbow and shoulder strength and range of motion after amputation. Also, an asymmetric body shape in the shoulder and upper arm area remained due to atrophic muscles still being present after elective amputation of the arm/hand and its bionic substitution. Therefore, the changes in body image observed in this study may primarily originate from the changed appearance of the hand – changing from a motionless atrophic appendix to a functional prosthetic limb. As summarized in Table 2, in five out of six patients *negative body evaluation* improved with bionic reconstruction, which was statistically significant. This means that it was easier for them to cope with their physical imperfections and that they had fewer negative associations with their body. The fact that modern prostheses resemble the appearance of a healthy hand more than an atrophic “plexus hand” (see Figure 1) might explain these results. Wearing a prosthesis might thereby reduce unwanted attention toward the appearance of the hand, which some of our patients described as bothersome and incriminating. Restoring cosmetic appearance has been described as an important factor for a positive body image and social comfort in amputees (Desteli et al., 2014). In line with this, a qualitative study in individuals with traumatic amputations has shown that having a prosthetic device helped them to minimize their sense of difference and was therefore perceived as very helpful in social situations (Saradjian et al., 2008). This social function of having a prosthesis was also verbalized by P1 who preferred not to use his prosthesis in daily life, but enjoyed wearing it at social gatherings. His otherwise limited use can be explained by the fact that the patient reported very little functional problems in daily life within the DASH questionnaire even before bionic reconstruction, resulting in a limited need for functional improvements with the prosthetic device. In line with this, five of six patients showed improvements or no changes with bionic reconstruction when asked whether their upper limb problems interfered with their social activities (question 22 in DASH). Three of them felt more capable or confident in relation to their upper limb impairment (question 30 in DASH), while the others had unchanged results.

In terms of prosthetic embodiment, major inter-individual differences were observed. For the statement “I felt the prosthesis only as a tool and not as a part of my body,” two participants did not agree at all (rating of 0 and 1/10), while the others rated this statement with 5, 6, 7, and 8, respectively. As expected, ratings of each individual for the statement “I had the feeling that the prosthesis was part of my body” were inverted to these results, although intra-group differences were not articulated that explicitly. Notably, the two individuals (P1 and P6) who had the strongest perception that their prosthesis was not a mere tool, but rather a part of their body, also enjoyed wearing it and felt that it looked very real. They were feeling that they had good control over their prosthetic device. Additionally, these two individuals had the highest scores for vital body dynamics and the lowest scores for negative body evaluation. This is in line with previous research in lower limb amputees describing a negative relation between body image disturbance and prosthesis satisfaction (Murray and Fox, 2002). Also, the BIQ-20 outcome and the embodiment of the prosthesis did not seem to be determined by the amputation level in our cohort, given that P1 had a transradial amputation and P6 had a glenohumeral amputation. The two individuals who perceived their prosthesis as a tool rather than a hand (P3 and P4, both with a transradial amputation) described it as not looking real. Perceived control and how they liked wearing the prosthesis varied between these two individuals. When asked whether they performed bimanual tasks with their intact hand/arm together with their prosthesis, five of six patients rated this with “5” or “4,” while P6 had a rating of “0.” Here, it is contra-intuitive that this participant who strongly perceived his prosthesis as a body part does not seem to use it at all together with his healthy arm, which would be expected for a prosthesis being integrated in the body scheme. A speculative explanation for this might be that his pain increased after amputation when performing activities, which may be pain in the unstable shoulder. Together with the general high pain level and perception of the arm as stiff and weak (DASH questions 24–29), this might have prevented him from doing bimanual tasks.

When putting the motivations and expectations of patients before bionic reconstruction in context with the outcomes, an interesting finding is that the two individuals who had the highest expectations for prosthetic function (P1 and P6) had the best outcomes in the BIQ-20, as well as enjoyed wearing their prosthesis most and perceived it as a part of their body. This gives the impression of a self-fulfilling prophecy for these two, while making it unlikely that less embodiment of the prosthesis and less clear findings in the BIQ-20 in the other patients can be explained by their expectations not being met. In this regard, however, it needs to be noted that expectations management is part of our pre-surgical procedure, which ensures that every patient gets a realistic understanding of possibilities and limitations. Another interesting finding was that half of the patients perceived their “plexus arm” as bothersome and hindering in performing ADLs. Surprisingly, the two patients without any useful elbow function were not amongst them. While all patients aimed for improved function with a bionic prosthesis, it is possible that they would have also benefited from an amputation and fitting with a cosmetic device. Indeed, a retrospective study including nine patients with an elective amputation after pan-plexus injury who wore no or only a cosmetic prosthesis, still found satisfactory outcomes and reduced shoulder pain (Maldonado et al., 2016). Still, our study procedure included the aim for restoring active hand function, and is thus not suitable for the evaluation of possible benefits of amputation without a functional prosthetic fitting.

In summary, our findings regarding prosthetic embodiment indicate that each individual perceives their prosthesis in a unique way. This is also in line with a recent qualitative study that investigated prosthetic embodiment and psychosocial implications in three upper limb amputees with a bi-directional interface enabling feedback (Middleton and Ortiz-Catalan, 2020), who all described their prosthetic embodiment differently. Another recent study identified an improved prosthetic embodiment over time when using sensory feedback (Cuberovic et al., 2019). Therefore, it is hard to predict the possible impact of using a bi-directional interface for prosthetic control in our group of patients. Similarly, we are not able to state whether the reason for amputation (elective vs. traumatic amputation) changes the way a prosthesis is perceived regarding the body image of a person.

While this study is the first to give insights in the long-term body perception and prosthetic embodiment of people with bionic reconstruction after brachial plexus injury, the small sample size limits the scientific significance of our observations. The limited cohort size, however, results from the novelty of the approach and the very limited number of individuals receiving bionic reconstruction world-wide. Still, neither the sample size nor the study design allows definite conclusions on whether bionic reconstruction should be used for improving negative body evaluation in patients with brachial plexopathy.

Furthermore, given the highly elective nature of the procedure, the results cannot be generalized for the whole population of patients with severe brachial plexus injuries. Patients, who after careful deliberation opt for keeping their functionless and asensate hand, likely have completely different motivations compared to the population we studied. Reasons for not undergoing bionic reconstruction may include concerns regarding a changed appearance after amputation, or a less positive attitude regarding prosthetic devices. Future qualitative studies might be able to better describe the viewpoints and priorities of patients (Graczyk et al., 2019). Conducting interviews with patients undergoing bionic reconstruction or deciding against it, might further aid an in-depth understanding of beliefs, mental processes, expectations and body image concerns related to decision-making and how they influence outcomes. Expanding our understanding of this topic will be helpful to determine how individuals feel and cope with their anatomy being replaced by technological tools.

## Data Availability Statement

The original contributions presented in the study are included in the article/Supplementary Material, further inquiries can be directed to the corresponding author.

## Ethics Statement

The studies involving human participants were reviewed and approved by Ethikkommission der Medizinischen Universität Wien Borschkegasse 8b/E06 1090 Wien. The patients/participants provided their written informed consent to participate in this study. Written informed consent was obtained from the individuals for the publication of any potentially identifiable images or data included in this article.

## Author Contributions

AS, LH, OA, and AP conceived and designed the study. AB, AS, LH, and CG performed the data acquisition. AB and AS analyzed the data. AS, IV, DF, and OP interpreted the data. AS, IV, LH, and CG had the main responsibility for writing the manuscript. All authors were involved in editing the manuscript and gave their final approval for publication.

## Conflict of Interest

The authors declare that the research was conducted in the absence of any commercial or financial relationships that could be construed as a potential conflict of interest.
