# Supplementary material for: Prosthetic Embodiment and Body Image Changes in Patients Undergoing Bionic Reconstruction Following Brachial Plexus Injury
Source: Front Neurorobot. 2021 Apr 30;15:645261. doi: 10.3389/fnbot.2021.645261 (PMC8119996; doi:10.3389/fnbot.2021.645261)
Supplement: Supplementary file 1 [file Table_1.DOCX]

Supplementary Material – Raw Data of Body Image Questionnaire-20

# Raw Data Body Image Questionnaire-20 before intervention

| Questions BIQ-20 | P1 | P2 | P3 | P4 | P5 | P6 |
| --- | --- | --- | --- | --- | --- | --- |
| 1. Overall I perceive myself as robust and strong. | 4 | 3 | 5 | 4 | 4 | 3 |
| 2. There is something wrong with my looks/appearance. | 2 | 2 | 4 | 5 | 1 | 2 |
| 3. I am healthy. | 5 | 1 | 3 | 4 | 1 | 3 |
| 4. Sometimes I feel boundless energy inside me. | 2 | 3 | 3 | 3 | 4 | 4 |
| 5. I can live well with my bodily defects/imperfectness.* | 1 | 1 | 2 | 2 | 4 | 1 |
| 6. My body bothers me frequently. | 5 | 5 | 3 | 5 | 2 | 1 |
| 7. I feel full of strength. | 5 | 2 | 3 | 3 | 2 | 4 |
| 8. I am dissatisfied with my figure (physique). | 2 | 3 | 4 | 4 | 1 | 1 |
| 9. Frequently I feel full of erotic tension. | 2 | 1 | 3 | 3 | 2 | 5 |
| 10. I wish for someone else’s body. | 1 | 1 | 2 | 5 | 1 | 1 |
| 11. Sometimes I feel disgusted toward my own body. | 1 | 2 | 2 | 3 | 1 | 1 |
| 12. I have enough powerful tension inside me. | 5 | 3 | 4 | 3 | 4 | 5 |
| 13. I do not like myself on photographs. | 2 | 2 | 2 | 5 | 2 | 4 |
| 14. I feel top fit. | 5 | 1 | 3 | 3 | 1 | 4 |
| 15. My bodily flaws do bother me a lot. | 3 | 1 | 3 | 4 | 2 | 1 |
| 16. I enjoy dealing with my sexual desires. | 1 | 3 | 4 | 4 | 2 | 5 |
| 17. I am physically capable of doing many things. | 5 | 4 | 3 | 4 | 4 | 5 |
| 18. Sometimes I wish to myself to look totally different. | 1 | 1 | 2 | 5 | 1 | 2 |
| 19. I feel at home in my body.* | 1 | 1 | 3 | 4 | 1 | 1 |
| 20. To dance frisky is of great fun to me. | 3 | 3 | 4 | 2 | 2 | 5 |
| **total score NBE** | **19** | **19** | **27** | **42** | **16** | **15** |
| **total score VBD** | **37** | **24** | **35** | **33** | **26** | **43** |

* reversed scores for these questions; BIQ-20= Body Image Questionnaire-20; NBE= negative body evaluation; VBD= vital body dynamics
scoring: 1 = is NOT true; 2 = is hardly true, 3 = is partly true; 4 = is substantially true, 5 = is COMPLETELY true

# Raw Data Body Image Questionnaire-20 after intervention

| Questions BIQ-20 | P1 | P2 | P3 | P4 | P5 | P6 |
| --- | --- | --- | --- | --- | --- | --- |
| 1. Overall I perceive myself as robust and strong. | 5 | 3 | 3 | 3 | 3 | 5 |
| 2. There is something wrong with my looks/appearance. | 1 | 3 | 3 | 4 | 4 | 1 |
| 3. I am healthy. | 5 | 2 | 2 | 4 | 4 | 5 |
| 4. Sometimes I feel boundless energy inside me. | 3 | 3 | 1 | 2 | 3 | 2 |
| 5. I can live well with my bodily defects/imperfectness.* | 1 | 1 | 1 | 3 | 2 | 1 |
| 6. My body bothers me frequently. | 1 | 1 | 1 | 4 | 2 | 1 |
| 7. I feel full of strength. | 5 | 3 | 2 | 2 | 3 | 4 |
| 8. I am dissatisfied with my figure (physique). | 1 | 2 | 2 | 5 | 1 | 1 |
| 9. Frequently I feel full of erotic tension. | 3 | 1 | 3 | 3 | 2 | 5 |
| 10. I wish for someone else’s body. | 1 | 1 | 1 | 2 | 1 | 1 |
| 11. Sometimes I feel disgusted toward my own body. | 1 | 1 | 1 | 2 | 1 | 1 |
| 12. I have enough powerful tension inside me. | 3 | 3 | 3 | 3 | 3 | 5 |
| 13. I do not like myself on photographs. | 1 | 4 | 1 | 4 | 2 | 3 |
| 14. I feel top fit. | 5 | 2 | 2 | 2 | 3 | 5 |
| 15. My bodily flaws do bother me a lot. | 1 | 1 | 1 | 3 | 3 | 1 |
| 16. I enjoy dealing with my sexual desires. | 1 | 3 | 5 | 4 | 2 | 5 |
| 17. I am physically capable of doing many things. | 5 | 4 | 3 | 3 | 4 | 5 |
| 18. Sometimes I wish to myself to look totally different. | 1 | 1 | 1 | 2 | 1 | 1 |
| 19. I feel at home in my body.* | 1 | 1 | 1 | 3 | 1 | 1 |
| 20. To dance frisky is of great fun to me. | 5 | 4 | 3 | 2 | 2 | 5 |
| **total score NBE** | **10** | **16** | **13** | **32** | **18** | **12** |
| **total score VBD** | **40** | **28** | **27** | **28** | **29** | **46** |

* reversed scores for these questions; BIQ-20= Body Image Questionnaire-20; NBE= negative body evaluation; VBD= vital body dynamics
scoring: 1 = is NOT true; 2 = is hardly true, 3 = is partly true; 4 = is substantially true, 5 = is COMPLETELY true
