# Supplementary material for: Prosthetic Embodiment and Body Image Changes in Patients Undergoing Bionic Reconstruction Following Brachial Plexus Injury
Source: Front Neurorobot. 2021 Apr 30;15:645261. doi: 10.3389/fnbot.2021.645261 (PMC8119996; doi:10.3389/fnbot.2021.645261)
Supplement: Supplementary file 2 [file Table_2.DOCX]

| **Patient ID** | P 1 | P 2 | P 3 | P 4 | P 5 | P 6 |
| --- | --- | --- | --- | --- | --- | --- |
| **Prosthesis wearing time in the last week** | Not within the last week (only for social events) | Less than twice per week | Every second day | Almost daily | Almost daily | Almost daily |
| **I had the feeling that the prosthesis was part of my body** | 7 | 5 | 5 | 6 | 4 | 8 |
| **I felt the prosthesis only as a tool, and not as a part of my body** | 1 | 5 | 7 | 8 | 6 | 0 |
| **I did bimanual tasks with my intact arm/hand together with my prosthesis** | 5 | 5 | 4 | 5 | 5 | 0 |
| **I felt that I had full control over the prosthesis** | 7 | 5 | 5 | 8 | 4 | 8 |
| **I liked wearing the prosthesis** | 9 | 4 | 3 | 8 | 5 | 10 |
| **I felt that my prosthesis looked like a real part of the body** | 10 | 7 | 2 | 3 | 4 | 9 |

Supplementary Material – Numeric values of all answers given in regard to prosthetic embodiment and prosthesis wearing time

Participants used a rating scale from 0-10 (0 never, 10 all the time).
