# Supplementary material for: Prosthetic Embodiment and Body Image Changes in Patients Undergoing Bionic Reconstruction Following Brachial Plexus Injury
Source: Front Neurorobot. 2021 Apr 30;15:645261. doi: 10.3389/fnbot.2021.645261 (PMC8119996; doi:10.3389/fnbot.2021.645261)
Supplement: Supplementary file 3 [file Table_3.DOCX]

Supplementary Material – Raw Data of Disabilities of the Arm, Shoulder and Hand (DASH) before and after intervention

| **Questions DASH** | **P1** | | **P2** | | **P3** | | **P4** | | **P5** | | **P6** | |
| --- | --- | --- | --- | --- | --- | --- | --- | --- | --- | --- | --- | --- |
|  | **pre** | **post** | **pre** | **post** | **pre** | **post** | **pre** | **post** | **pre** | **post** | **pre** | **post** |
| 1. Open a tight or new jar. | 2 | **1** | 3 | **1** | 4 | **2** | 3 | **2** | 3 | **2** | 2 | 2 |
| 2. Write. | 1 | 1 | 3 | **1** | 3 | 4 | 1 | 1 | 4 | 4 | 3 | **2** |
| 3. Turn a key. | 1 | 1 | 1 | 1 | 1 | 1 | 1 | 1 | 2 | 2 | 1 | 1 |
| 4. Prepare a meal. | 2 | **1** | 3 | **2** | 3 | **2** | 3 | 3 | 2 | 2 | 4 | **2** |
| 5. Push open a heavy door. | 1 | 1 | 3 | **1** | 2 | **1** | 1 | 2 | 2 | **1** | 2 | **1** |
| 6. Place an object on a shelf above your head. | 1 | 1 | 4 | **1** | 2 | 2 | 3 | 4 | 2 | 2 | 5 | **4** |
| 7. Do heavy household chores (e.g., wash walls, wash floors). | 1 | 1 | 5 | **2** | 5 | **2** | 3 | **2** | 2 | 3 | 4 | **2** |
| 8. Garden or do yard work. | 2 | **1** | 3 | **1** | 4 | **3** | 3 | 3 | 2 | 2 | 4 | **2** |
| 9. Make a bed. | 1 | 1 | 2 | 2 | 3 | **2** | 3 | 3 | 3 | 3 | 3 | **2** |
| 10. Carry a shopping bag or briefcase. | 2 | **1** | 2 | **1** | 3 | **2** | 1 | 2 | 2 | **1** | 4 | **2** |
| 11. Carry a heavy object (over 10lbs/5kg). | 1 | 1 | 4 | **1** | 3 | **2** | 1 | 2 | 2 | 2 | 4 | **3** |
| 12. Change a lightbulb overhead. | 1 | 1 | 5 | **4** | 4 | **3** | 3 | 3 | 2 | 2 | 4 | 4 |
| 13. Wash or blow dry your hair. | 1 | 1 | 2 | **1** | 2 | **1** | 1 | 1 | 2 | 3 | 1 | 1 |
| 14. Wash your back. | 1 | 1 | 4 | **3** | 4 | **2** | 2 | 2 | 3 | 3 | 3 | **2** |
| 15. Put on a pullover sweater. | 1 | 1 | 4 | **1** | 4 | **2** | 2 | 2 | 3 | 3 | 2 | **1** |
| 16. Use a knife to cut food. | 1 | 1 | 5 | **3** | 5 | 5 | 3 | 4 | 4 | 4 | 4 | **2** |
| 17. Recreational activities which require little effort (e.g., cardplaying, knitting, etc.). | 2 | 2 | 2 | **1** | 2 | **1** | 1 | 3 | 2 | 3 | 3 | **1** |
| 18. Recreational activities in which you take some force or impact through your arm, shoulder or hand (e.g., golf, hammering, tennis, etc.). | 2 | 3 | 4 | **1** | 5 | **4** | 3 | 3 | 4 | 4 | 4 | **2** |
| 19. Recreational activities in which you move your arm freely (e.g., playing frisbee, badminton, etc.). | 1 | 1 | 5 | **1** | 3 | **2** | 1 | 2 | 3 | 4 | 4 | **2** |
| 20. Manage transportation needs (getting from one place to another). | 1 | 1 | 1 | 1 | 3 | **1** | 1 | 1 | 1 | 2 | 1 | 1 |
| 21. Sexual activities. | 2 | **1** | 4 | 4 | 3 | **1** | 2 | 2 | 4 | 4 | 1 | 1 |
| 22. During the past week, to what extent has your arm, shoulder or hand problem interfered with your normal social activities with family, friends, neighbours or groups? | 1 | 1 | 3 | 4 | 3 | **1** | 4 | **3** | 3 | 3 | 2 | **1** |
| 23. During the past week, were you limited in your work or other regular daily activities as a result of your arm, shoulder or hand problem? | 2 | **1** | 4 | **3** | 4 | **1** | 4 | **3** | 3 | 3 | 2 | **1** |
| 24. Severity of arm, shoulder or hand pain (in the last week). | 1 | 2 | 4 | 5 | 5 | **2** | 5 | 5 | 4 | **3** | 5 | **4** |
| 25. Severity of arm, shoulder or hand pain when you performed any specific activity (in the last week). | 1 | 1 | 1 | 3 | 5 | **3** | 5 | 5 | 3 | 3 | 3 | 4 |
| 26. Severity of tingling (pins and needles) in your arm, shoulder or hand (in the last week). | 1 | 2 | 4 | 5 | 5 | **2** | 1 | 3 | 4 | **3** | 5 | **4** |
| 27. Severity of weakness in your arm, shoulder or hand (in the last week). | 1 | 1 | 5 | 5 | 5 | **4** | 5 | **3** | 5 | **3** | 1 | 4 |
| 28. Severity of stiffness in your arm, shoulder or hand (in the last week). | 2 | 2 | 5 | **4** | 3 | 4 | 5 | 5 | 5 | **3** | 4 | 4 |
| 29. During the past week, how much difficulty have you had sleeping because of the pain in your arm, shoulder or hand? | 1 | 1 | 2 | 3 | 4 | **2** | 3 | **2** | 3 | 3 | 3 | 3 |
| 30. I feel less capable, less confident or less useful because of my arm, shoulder or hand problem. | 2 | **1** | 5 | **4** | 4 | **3** | 5 | 5 | 3 | 3 | 1 | 1 |
| **total DASH Score*** | **8.3** | **5** | **60** | **33.3** | **63.3** | **30.8** | **40.8** | **43.3** | **47.5** | **44.2** | **49.2** | **30** |

For all questions, “1” means no difficulty or limitation, while “5” means “unable to perform the task”, or “extreme pain/sleep difficulties” or “strong agreement regarding reduced confidence”. In the post-amputation DASH values, a light blue background with white letters indicates an improvement of one point, a dark blue background an improvement of two or more points. Similarly, a light yellow background indicates worse values of one point, while for two or more points a darker yellow background is used.
